# Supplementary material for: Ultra‐high temporal resolution 4D angiography using arterial spin labeling with subspace reconstruction
Source: Magn Reson Med. 2024 Dec 29;93(5):1924–41. doi: 10.1002/mrm.30407 (PMC11893029; doi:10.1002/mrm.30407)
Supplement: Supplementary file 1 — Figure S1. Angiograms reconstructed by Extreme MRI with different regularization weights. Low regularization weight kept the fidelity of angiography but lost SNR, whereas high regularization weight overly smoothed the image and lost visibility of thin vessels, as indicated by the yellow circles. Arteries pointed by the red arrows also appeared at late timepoints only at high regularization weight, confirming these were artifacts introduced due to over‐regularized on the temporal dimension. Figure S2. Angiography reconstructed by “LLR‐matched” with different regularization weights. In a similar way to Extreme MRI, the choice of regularization weight for LLR matched was a compromise between excessive smoothing and noisy images. Figure S3. An example movie of dynamic MRA reconstructed by the subspace method shows smooth blood flow into the brain. The contrast of each frame was separately adjusted for clarity. From left to right: Maximum intensity projection (MIP) in Sagittal, coronal and transverse view. Figure S4. The 12 coefficient maps corresponding to 12 extracted principal components. Table S1. Acquisition parameters for the three different in vivo protocols. [file MRM-93-1924-s001.docx]

Supplementary material for “Ultra-high temporal resolution 4D angiography using arterial spin labeling with subspace reconstruction”

| **Label** | Labeling duration | $1.8 s$ |
| --- | --- | --- |
|  | RF pulse type | Gaussian |
|  | RF flip angle | $20^{\circ}$ |
|  | RF pulse duration | $500 \mu$s |
|  | RF pulse separation | $1000 \mu$s |
|  | Mean tagging gradient | $0.8 mT/m$ |
|  | Tagging gradient amplitude | $6 mT/m$ |

Table 1. Acquisition parameters for the three different in vivo protocols.


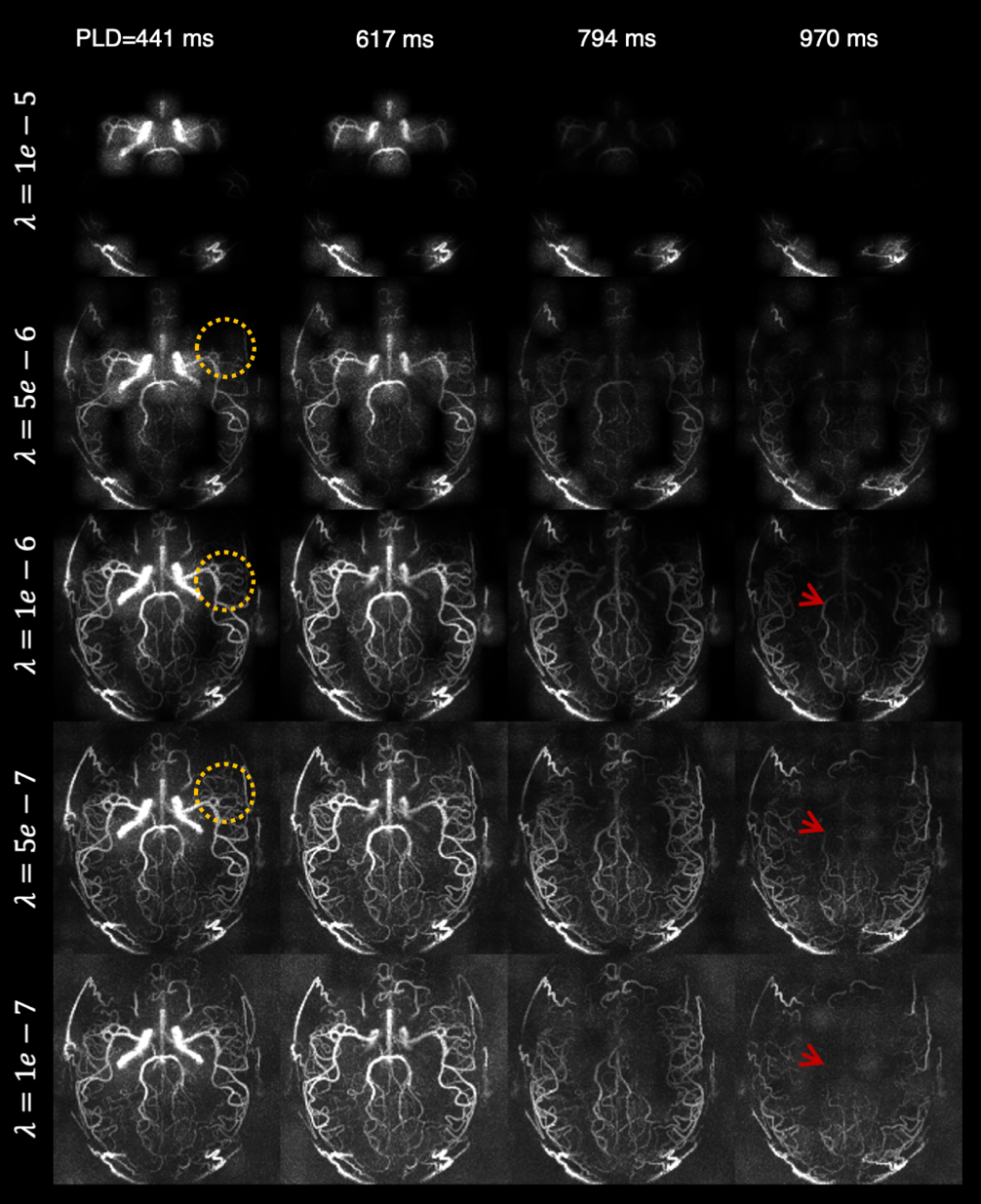


Figure S1. Angiograms reconstructed by Extreme MRI with different regularization weights. Low regularization weight kept the fidelity of angiography but lost SNR, whereas high regularization weight overly smoothed the image and lost visibility of thin vessels, as indicated by the yellow circles. Arteries pointed by the red arrows also appeared at late timepoints only at high regularization weight, confirming these were artifacts introduced due to over-regularized on the temporal dimension.


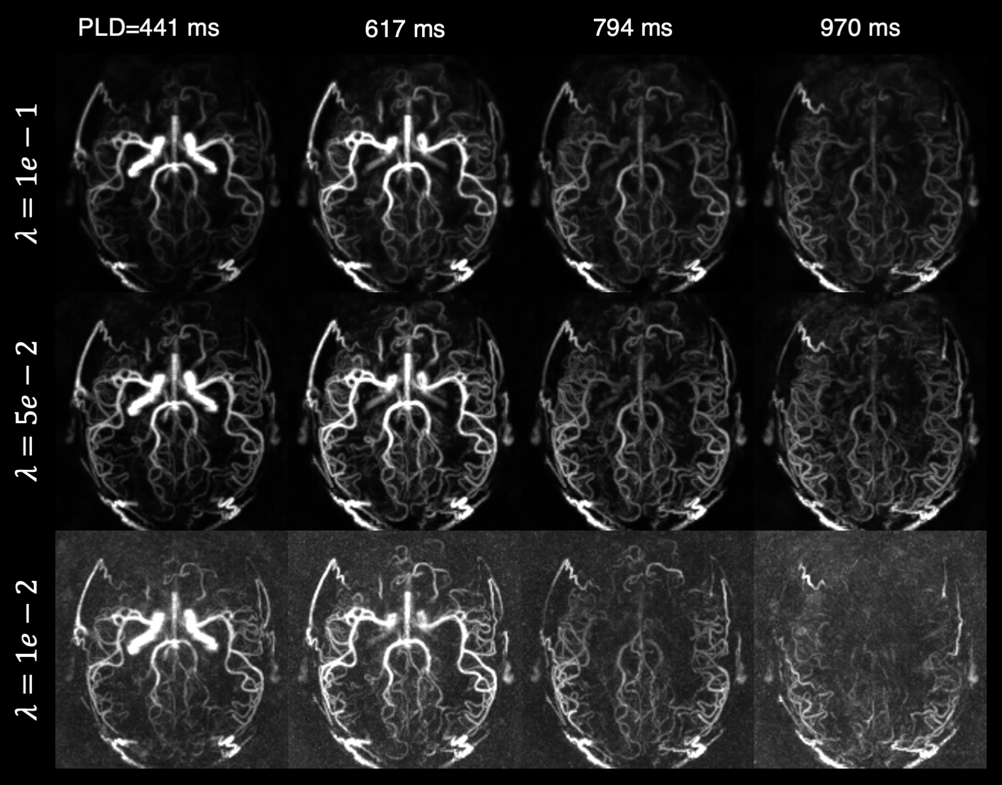


Figure S2. Angiography reconstructed by “LLR-matched” with different regularization weights. In a similar way to Extreme MRI, the choice of regularization weight for LLR matched was a compromise between excessive smoothing and noisy images.


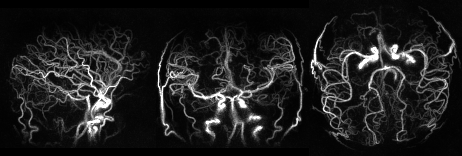


Figure S3. Example movie of dynamic MRA reconstructed by the subspace method showing smooth blood flow into the brain. The contrast of each frame was separately adjusted for clarity. From left to right: Maximum intensity projection (MIP) in Sagittal, coronal and transverse view.


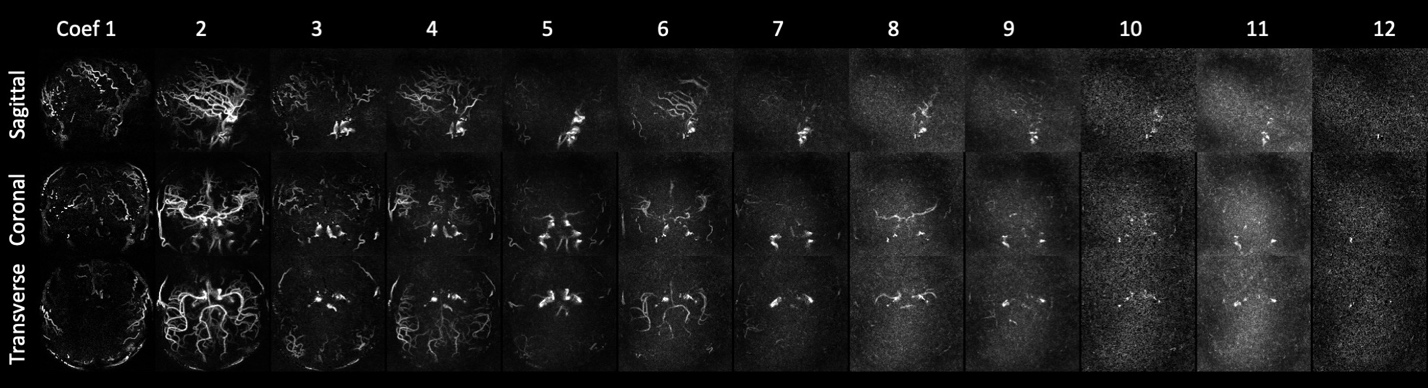


Figure S4. The 12 coefficient maps corresponding to 12 extracted principal components.
